# Predictors of contact with services for mental health problems among children with comorbid long-term physical health conditions: a follow-up study

**DOI:** 10.1007/s00787-022-02105-4

**Published:** 2022-11-10

**Authors:** Laura Panagi, Simon R. White, Charlotte Howdle, Sophie Bennett, Isobel Heyman, Roz Shafran, Tamsin Ford

**Affiliations:** 1 Department of Psychiatry, University of Cambridge, The Clifford Allbutt Building, Biomedical Innovation Hub/Bay 13, Cambridge Biomedical Campus, Hills Road, Cambridge, CB2 OAH UK; 2 School of Clinical Medicine, University of Cambridge, Addenbrooke’s Hospital, Hills Road, Cambridge, CB2 0SP UK; 3 UCL Great Ormond Street Institute of Child Health, 30 Guilford Street, London, WC1N 1EH UK

**Keywords:** Children, Mental health, Physical health, Predictors, Service contact, Follow-up

## Abstract

Children with long-term physical health conditions (pLTCs) are at increased risk of developing mental health comorbidities, although most do not access services for their mental health. No previous studies have examined the determinants of contact with services for mental health concerns among this group of children. This 3-year longitudinal study involved a population-based sample of children aged 5–16 years from the British Child and Adolescent Mental Health Surveys conducted in 1999 and 2004. In children with comorbid pLTCs and mental health disorders at baseline (*N* = 397), we examined associations between several child-, family- and service-related factors and (a) contact with primary health care, (b) contact with paediatrics and (c) contact with child and adolescent mental health services over 3-year follow-up (2002 and 2007). Separate multivariable binary logistic regressions were conducted for each service. The impact of mental health difficulties on the child and contact with the teacher predicted contact with all three services. Adolescent age, female gender, larger family size, some or marked academic difficulties, and having parents with educational qualification(s) were specific predictors of contact with primary health care. Male gender, stressful life events, and contact with primary health care were specific predictors of contact with child and adolescent mental health services. No other factors predicted contact with paediatrics. Our findings highlight the role of child-, family-, and service-related factors in accessing mental health care in children with comorbid pLTCs and mental health disorders which could inform planning and provision of services to reduce unmet mental health needs.

**Supplementary Information:**

The online version contains supplementary material available at 10.1007/s00787-022-02105-4.

## Introduction

Children with long-term physical health conditions (pLTCs) are at two to four times greater risk of developing mental health disorders as compared to their physically healthier counterparts [1]. Left untreated, mental health disorders predict adverse health outcomes [2] and produce increased healthcare costs [3]. Nevertheless, only a quarter of children attending paediatric clinics who had a probable psychiatric disorder received specialist help from child and adolescent mental health services [4]. Outcomes for referrals to child and adolescent mental health services in the UK for emotional difficulties relating to physical health conditions are unknown for almost 80% of young patients [5]. Understanding predictors of service contact would help clinicians and researchers identify targeted approaches to address children’s unmet needs.

Existing research on contact with services for mental health concerns in children of the general population is predominantly cross-sectional and has mostly focussed on specialist mental health services. Results from studies examining age [6–9], sex [6, 10–12], and socio-economic status [8, 9, 11, 13] are mixed, while caregiver educational level has not been linked to service use [6, 12, 14]. There is also conflicting evidence as to whether ethnicity influences help-seeking [6, 13, 15]. A recent systematic review reported that being from the dominant ethnic group in the United States is a significant predictor of service contact [14].

Experiencing stressful life events [10], academic difficulties [12] and poor general health [16] have been associated with greater service contact in children, as well as the recognition of mental health difficulties by parents [17] and teachers [10]. In addition, previous studies found that contact with services is predicted by greater impact of mental health difficulties on the child [10] and their parents [14]. The area of residence (south vs. north of Britain) [10], larger family size [10], parental mental distress [12], and being a single parent [12] have all also been related to contact with services for child’s mental health.

Most of previous studies were conducted outside the UK [6–9, 11, 12, 14, 15]. An advantage of using a UK population to explore predictors of service contact is that services are freely available in the UK, meaning that affordability or having health insurance should not influence access. It is also important to investigate contact with different providers, especially in children with pLTCs who are regularly seen by primary health care staff or paediatricians. A previous population-based study of mental health-related service access in the UK reported that contact with teachers in relation to mental health concerns, and contact with primary health care professionals both predicted contact with paediatrics and specialist mental health services [10]. Others have demonstrated that contact with paediatrics predicts contact with mental health services [18].

**Fig. 1:**
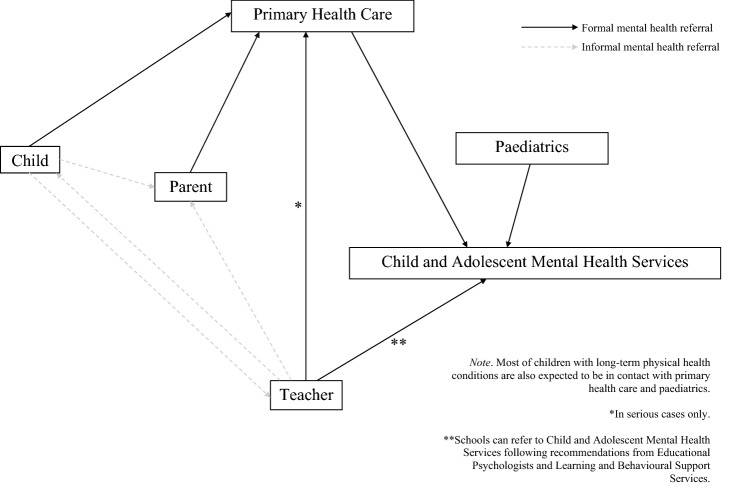
Pathways to specialist mental health services for children and young people in the UK

Indeed, the pathways into specialist mental health care for children in the UK are complex (Fig. 1), particularly for children with multiple conditions. For example, primary health care professionals may diagnose and manage mental health problems in children and young people (CYP), but they should make appropriate referrals if problems are complex and/or persistent. The wider practice team has an important role in promoting good mental health (e.g. good sleep, exercise, healthy eating, and education) [19]. Paediatricians should also be able to assess CYP by considering the biological, psychological and social factors contributing to the presentation of mental health problems. They should be promoting positive mental health and make appropriate referrals for mental health problems in the CYP they see. Community paediatricians frequently take a lead role on the assessment and management of neurodevelopmental disorders such as Autism Spectrum Disorder and Attention-Deficit Hyperactivity Disorder [20]. CYP whose problems are primarily school-based need to receive input from school resources in the first instance (e.g. Educational Psychologists and Learning and Behavioural Support Services), before any referral can be accepted by specialist mental health services [21]. None of the previous studies have investigated predictors of contact with different health care services for mental health concerns in children with comorbid pLTCs and psychopathology.

The aim of this study is to describe service contact among a UK population-based sample of children with comorbid pLTCs and mental health disorders and to investigate predictors of contact with different services in relation to their mental health over 3 years.

## Methods

### Participants and procedure

We used data from the British Child and Adolescent Mental Health Surveys (BCAMHS), which comprise two comparable population-based surveys of school-aged children 5–16 years, conducted in the UK in 1999 and 2004 (*N* = 18,403). Detailed survey design information is reported in the online resource 1. The two baseline samples, which are mutually exclusive, were followed up with a repeat survey in 2002 and 2007, respectively. Informed consent was obtained from legal guardians (for children < 11 years old) and from young participants. The original BCAMHS were approved by the Medical Research Ethics Committees [2]. Specific ethical approval was not necessary for this study as no additional participant contact was required for this secondary analysis; the data are available via application to the UK Data Service [22].

**Fig. 2:**
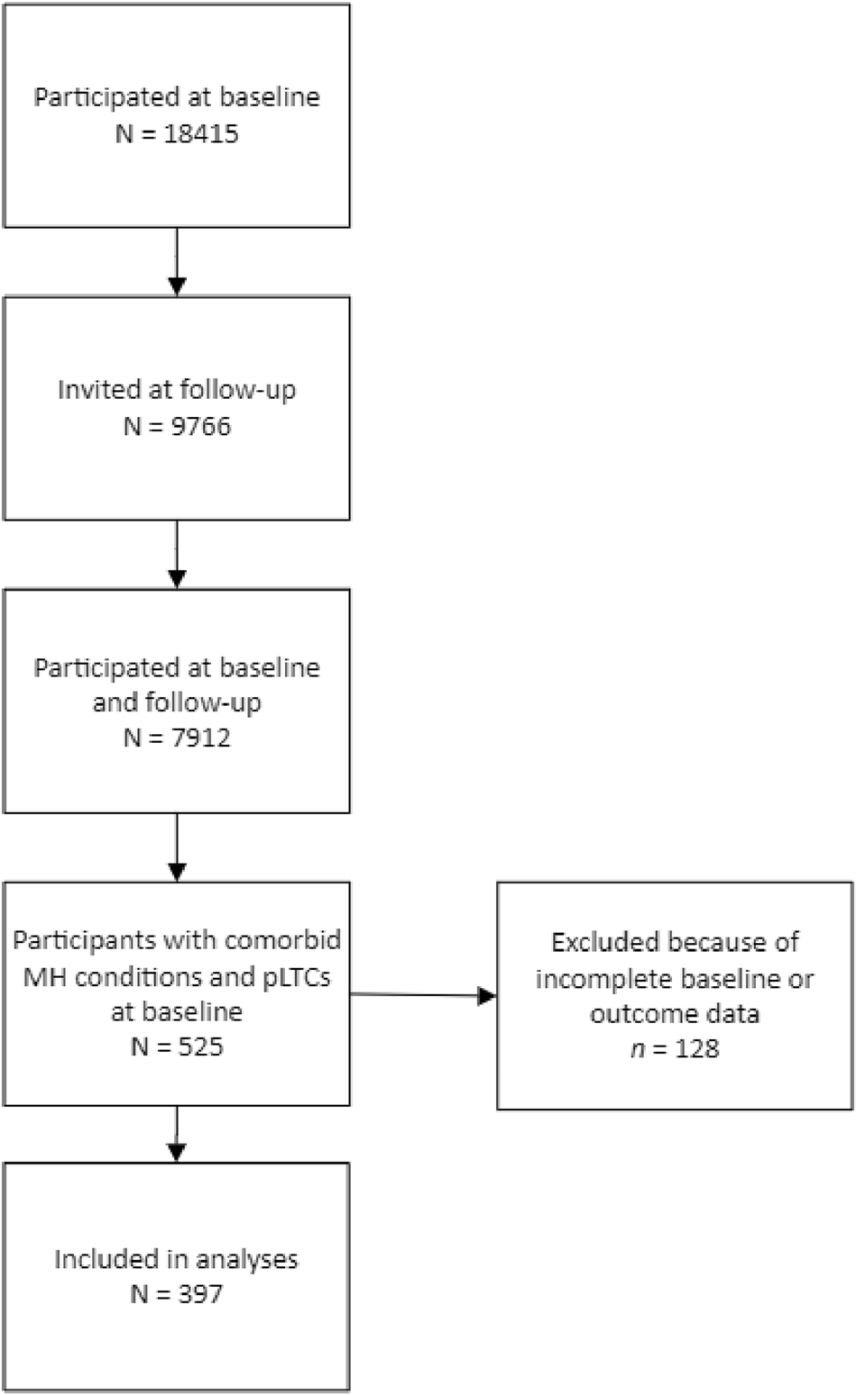
Study flow diagram

Data collection involved parents (the natural mother in 94% of cases), young people (aged ≥ 11 years) and teachers (the family nominated a teacher who knew the child best). Parents and young people were interviewed face-to-face by trained lay interviewers using computer-assisted interviews and self-reported questionnaires were also completed, while teachers were mailed a questionnaire. Figure 2 describes the sample selected for our analysis. We included children with at least one mental health disorder and at least one pLTC at baseline. Participants with incomplete data on study predictors or outcome measures were excluded (*n* = 128), resulting in an analytical sample of 397 participants.

Mental health disorders were assessed using the Development and Wellbeing Assessment (DAWBA) [23]. The DAWBA is a standardised diagnostic tool that combines highly structured questions about common childhood disorders with semi-structured probes, incorporating information from parents, young people (gathered via interview), and teachers (gathered via questionnaire). Computer-generated predictions of psychiatric disorders were reviewed by clinicians who assigned diagnoses (see online resource 2) based on the International Classification of Diseases-10 [24].

pLTCs were assessed using parent report on whether the child has any of the following conditions at time assessment (yes/no): asthma, eczema, hayfever, epilepsy, cerebral palsy, muscle disease, co-ordination problems, heart problems, food allergy, kidney/urinary tract problems, a condition present since birth (e.g. club foot or cleft palate), deformities, spina bifida, cystic fibrosis, blood disorders, missing limb(s), diabetes, cancer, vision problems, or hearing problems. This selection was based on the consensus definition of chronic health conditions in childhood [25] and agreed with our Study Steering Committee.

## Measures

### Predictor variables

Candidate predictors were selected based on previously detected associations with mental health-related contact with services. Child-level factors included age, gender, ethnicity, general heath level, number of stressful life events during lifetime and academic attainment reflecting difficulties with reading, spelling, or mathematics compared to peers. Child-level factors also included parental and teacher recognition of mental health difficulties, assessed using a question from the Strengths and Difficulties Questionnaire (SDQ) [26] ‘Overall, do you think that your/this child has difficulties in one or more of the following areas: emotions, concentration, behaviour or being able to get on with other people?’. The Impact score of the parental SDQ impact subscale was also used to measure the impact of mental health difficulties and comprises questions about the child’s distress (‘Do the difficulties upset or distress your child’) and participation in general activities (‘Do the difficulties interfere with your child's everyday life in the following areas: home life, friendships, classroom learning, leisure activities?’)*.* Impact scores range from 0 to 10 with higher scores indicating greater impact on the child. The internal consistency of the impact subscale was good in our sample (*α* = 0.78). Burden on the family was also measured using the SDQ question ‘Do the difficulties put a burden on you or the family as a whole’. We use the term mental health *difficulties* to refer to the SDQ responses and the term mental health *disorders* to refer to the DAWBA diagnoses throughout*.* A table listing the SDQ questions used in this study is included in the online resource 3. 

Family-level factors included housing tenure, parental educational qualification(s) from GCSE or equivalent, area of residence, family structure, number of siblings, and parental mental distress. Parental mental distress was indexed using the 12-item General Health Questionnaire [27]. Total scores range from 0 to 12 with higher scores indicating greater distress. A binary measure was created using a cut-off point of 3 [28]. The internal consistency of this measure was very good in our sample (*α* = 0.89).

Service-related factors were included as study predictors in the models as described below (Statistical analysis). The parent responded to the question ‘In the past year, have you, or [child’s name], been in contact with any of these people because of worries about his/her emotions, behaviour or concentration?’. We measured contact with teacher including head of year, head-teacher or special educational needs co-ordinator, contact with primary health care including general practitioner, family doctor or practice nurse, and contact with paediatric services including someone specialising in children’s physical health (for example a hospital or community paediatrician) in the previous year.

All predictor variables were measured at baseline, except contact with services which was measured at follow-up. All predictor variables were reported by the parent, except academic attainment and teacher recognition of mental health difficulties which were reported by the teacher.

### Outcome variables

Contact with services at follow-up involved a repetition of the question on contact with primary health care and paediatrics. An additional question was related to contact with child and adolescent mental health services including someone specialising in children’s mental health (for example child psychiatrist or child psychologist) over the preceding 12 months. Given the broad questions parents were asked, it is conceivable that contact with services might range from a telephone advice to multiple in-person therapeutic sessions.

### Statistical analysis

Differences between the analytic sample and those excluded from the analysis because of incomplete data were tested using independent samples *t* test and Chi-square tests. Sample characteristics at baseline and follow-up were summarised as numbers and percentages. Unadjusted associations between our candidate predictors and the three outcome measures were examined using binary logistic regression analyses.

In main analyses, we explored the independent effect of study predictors on contact with primary health care for mental health problems, contact with paediatrics for mental health problems, and contact with child and adolescent mental health services. Multivariable binary logistic regression analyses were carried out using backward stepwise method (likelihood ratio) where all variables are entered all together with the least statistically significant removed until all those remaining are statistically significant at a probability of 0.05. All significant predictors were then re-entered to a single-step multivariable binary logistic regression model along with age, sex, and housing tenure. This strategy was repeated for each service. Child- and family-level predictors were inserted to all three models. With regards to service-related predictors, teacher contact was inserted to all three models, contact with primary health care was inserted to the models predicting contact with paediatrics and child and adolescent mental health services, and contact with paediatrics was inserted to the model predicting contact with child and adolescent mental health services. In secondary analyses, age by gender interactions were added to the final, single-step logistic regression models. All analyses were conducted using SPPS v27.

## Results

Comparisons between the study sample and those excluded from the analysis because of incomplete data revealed that excluded children were more likely to be of older age (*phi* = − 0.10, *p* = 0.030), to have worse general health level (*phi* = − 0.12, *p* = 0.010), and to have experienced more stressful life events over their lifetime (*phi* = − 0.13, *p* = 0.006). Parents of excluded children were more likely to recognise their child as suffering from definite or severe mental health difficulties (*phi* = − 0.12, *p* = 0.007) with a greater impact on child’s distress levels and everyday life (*d* = 0.25, *p* = 0.012) and a greater burden to the family (*phi* = − 0.12, *p* = 0.007). No differences were observed between included and excluded participants in any other baseline or follow-up measure.

### Unadjusted associations with service contact

In unadjusted analyses, parental recognition of definite or severe mental health difficulties that have a greater impact on child’s distress levels and everyday life and add a greater burden to the family, as well as contact with the teacher predicted contact with all three services (Table 1). In addition, contact with primary health care predicted contact with paediatrics [OR = 4.11 (2.00, 8.45)] and child and adolescent mental health services [OR = 4.33 (2.49, 7.56)], and contact with paediatrics predicted contact with child and adolescent mental health services [OR = 3.47 (1.64, 7.33)]. Children with average or above average academic attainment were less likely to report contact with primary health care [OR = 0.50 (0.30, 0.86)] and children whose parents had educational qualification(s) were more likely to report contact with primary health care [OR 1.88 (1.05, 3.37)]. Parents who rented their house were less likely to report contact with paediatric services [OR = 0.44 (0.20, 0.94)] and boys were more likely to have been in contact with child and adolescent mental health services [OR = 0.30 (0.15, 0.57)].

**Table 1:** Sample characteristics at baseline and follow-up and unadjusted associations with the three outcome measures (*N* = 397)

Characteristics	*n* (%)	Contact with primary health care OR (95% CI)	Contact with paediatrics OR (95% CI)	Contact with child and adolescent mental health services OR (95% CI)
Age				
< 11 years old	207 (52.1)	1.33 (0.83, 2.14)	0.49 (0.23, 1.04)	0.72 (0.43, 1.23)
≥ 11 years old	190 (47.9)			
Gender				
Male	247 (62.2)	1.11 (0.69, 1.81)	0.89 (0.43, 1.85)	0.30 (0.15, 0.57)
Female	150 (37.8)			
Ethnicity				
White	377 (94.7)	0.82 (0.27, 2.50)	1.13 (0.25, 5.08)	0.49 (0.11, 2.17)
Other	21 (5.3)			
General health level				
Good or very good	307 (77.3)	1.18 (0.68, 2.05)	1.25 (0.56, 2.79)	0.86 (0.45, 1.64)
Fair, bad or very bad	90 (22.7)			
Number of stressful life events				
< 3	278 (70.0)	1.46 (0.88, 2.40)	0.70 (0.31, 1.59)	1.57 (0.91, 2.71)
≥ 3	119 (30.0)			
Educational attainment				
Some or marked difficulties	252 (63.5)	0.50 (0.30, 0.86)	0.51 (0.22, 1.15)	0.80 (0.46, 1.40)
Average or above average attainment	145 (36.5)			
Any parental qualifications				
No	113 (28.5)	1.88 (1.05, 3.37)	1.95 (0.79, 4.85)	1.13 (0.63, 2.03)
Yes	284 (71.5)			
Housing tenure				
Own	210 (52.9)	1.10 (0.68, 1.76)	0.44 (0.20, 0.94)	0.75 (0.44, 1.27)
Rent	187 (47.1)			
Area of living				
South Britain	183 (46.1)	0.82 (0.51, 1.31)	0.65 (0.32, 1.32)	0.95 (0.57, 1.61)
North Britain	214 (53.9)			
Number of siblings				
< 2	103 (25.9)	1.48 (0.83, 2.62)	1.39 (0.59, 3.29)	1.29 (0.69, 2.41)
≥ 2	294 (74.1)			
Family structure				
Traditional	212 (53.4)	1.13 (0.87, 1.46)	0.79 (0.52, 1.19)	0.99 (0.74, 1.32)
Blended	57 (14.4)			
Lone parent	128 (32.2)			
Parental mental distress [GHQ]				
Score < 4	252 (63.5)	1.27 (0.78, 2.06)	0.82 (0.39, 1.73)	1.47 (0.87, 2.50)
Score ≥ 4	145 (36.5)			
Recognition of mental health difficulties by the parent [P: SDQ question]				
No or minor difficulties	213 (53.7)	2.19 (1.35, 3.55)	3.56 (1.62, 7.85)	3.14 (1.79, 5.49)
Definite or severe	184 (46.3)			
Recognition of mental health difficulties by the teacher [T: SDQ question]				
No or minor difficulties	170 (42.8)	1.04 (0.65, 1.68)	1.08 (0.53, 2.20)	1.46 (0.85, 2.52)
Definite or severe difficulties	227 (57.2)			
Impact of mental health difficulties on the child [P: SDQ Impact score]	2.72 (2.76)	1.17 (1.08, 1.28)	1.27 (1.13, 1.43)	1.25 (1.14 1.37)
Mental health difficulties putting a burden on the family [P: SDQ question]				
Not at all or only a little	194 (48.9)	1.51 (0.94, 2.44)	2.13 (1.01, 4.49)	2.69 (1.53, 4.74)
Quite a lot or a great deal	203 (51.1)			
Contact with teacher at follow-up				
No	242 (61.0)	5.94 (3.52, 10.03)	10.91 (4.12, 28.88)	4.98 (2.82, 8.81)
Yes	155 (39.0)			
Contact with primary health care at follow-up				
No	309 (77.8)		4.11 (2.00, 8.45)	4.33 (2.49, 7.56)
Yes	88 (22.2)			
Contact with paediatrics at follow-up				
No	363 (91.4)			3.47 (1.64, 7.33)
Yes	34 (8.6)			
Contact with child and adolescent mental health services at follow-up				
No	329 (82.9)			
Yes	68 (17.1)			

Unadjusted odds ratios and 95% confidence intervals were tested using binary logistic regressions

*GHQ* general health questionnaire, *M* mean, *N* number, *n* number, *P* parental, *SD* standard deviation, *SDQ *strengths and difficulties questionnaire, *T* teacher

### Independent predictors of service contact

Table 2 presents independent predictors of contact with primary health care for child’s mental health. Results showed that older children [aOR = 2.09 (1.20, 3.66)], girls [aOR = 1.88 (1.05, 3.37)], and children with two or more siblings [aOR = 2.36 (1.18, 4.72)] had higher odds of mental health-related contact with primary health care professionals. Average or above average academic attainment [aOR = 0.45 (0.24, 0.84)] predicted decreased likelihood of reporting contact with primary health care whereas parental educational qualification(s) [aOR = 2.12 (1.07, 4.20)] increased the likelihood of reporting contact with primary health care. Greater impact of difficulties on child’s distress levels and everyday life [aOR = 1.11 (1.01, 1.22)], and contact with the teacher [aOR = 7.44 (4.11, 13.45)] were also predictors of contact with primary health care.

**Table 2:** Multivariable binary logistic regressions predicting contact with primary health care in children with comorbid long-term physical health conditions (*N* = 397)

	Contact with primary health care adjusted OR (95% CI)
Age [ref. cat. < 11 years old]	2.09 (1.20, 3.66)
Sex [ref. cat. male]	1.88 (1.05, 3.37)
Housing tenure [ref. cat. own]	1.21 (0.64, 2.31)
Educational attainment [ref. cat. some or marked difficulties]	0.45 (0.24, 0.84)
Family structure [ref. cat. traditional]	
Blended	0.71 (0.31, 1.62)
Lone	1.95 (0.97, 3.90)
Number of siblings [ref. cat. < two]	2.36 (1.18, 4.72)
Parental qualifications [ref. cat. no]	2.12 (1.07, 4.20)
Impact of mental health difficulties on the child [score]	1.11 (1.01, 1.22)
Contact with teacher at follow-up [ref. cat. no]	7.44 (4.11, 13.45)

Multivariable binary logistic regression was carried out using backward stepwise (likelihood ratio) method. All significant predictors where then entered to the final model (presented here) including age, sex and housing tenure

*CI* confidence interval, *N* number, *OR* odds ratio, *ref. cat.* reference category

Mental health-related contact with paediatrics was only predicted by greater impact of difficulties on child’s distress levels and everyday life [aOR = 1.21 (1.06, 1.38)] and contact with the teacher [aOR = 9.04 (3.31, 24.69)] (Table 3). The same pattern of results was observed for child and adolescent mental health services; increased impact [aOR = 1.17 (1.06, 1.30)] and teacher contact [aOR = 2.94 (1.53, 5.66)]. Other predictors specific to child and adolescent mental health services were male gender [fewer girls were seen; aOR = 0.31 (0.15, 0.64)], the experience of three or more stressful life events over the lifetime [aOR = 2.21 (1.15, 4.27)], and contact with primary health care [aOR = 2.86 (1.49, 5.52)] (Table 3).

**Table 3:** Multivariable binary logistic regressions predicting contact with paediatrics and child and adolescent mental health services in children with comorbid long-term physical health conditions (*N* = 397)

	^a^Contact with paediatrics adjusted OR (95% CI)	^b^Contact with child and adolescent mental health services adjusted OR (95% CI)
Age [ref. cat. < 11 years old]	0.54 (0.24, 1.21)	0.67 (0.36, 1.23)
Sex [ref. cat. male]	1.57 (0.69, 3.55)	0.31 (0.15, 0.64)
Housing tenure [ref. cat. own]	0.52 (0.23, 1.18)	0.70 (0.38, 1.29)
Number of stressful life events [ref. cat. < than 3]		2.21 (1.15, 4.27)
Impact of mental health difficulties on the child [score]	1.21 (1.06, 1.38)	1.17 (1.06, 1.30)
Contact with teacher at follow-up [ref. cat. no]	9.04 (3.31, 24.69)	2.94 (1.53, 5.66)
Contact with primary health care at follow-up [ref. cat. no]		2.86 (1.49, 5.52)

Multivariable binary logistic regressions were carried out using backward stepwise (likelihood ratio) method. All significant predictors where then entered to the final model (presented here) including age, sex, and housing tenure. Separate regressions were conducted for each service

*CI* confidence interval, *N* number, *OR* odds ratio, *ref. cat.* reference category

^a^The model predicting contact with paediatrics was adjusted for age, sex, housing tenure, impact of mental health difficulties on the child, and contact with teacher at follow-up

^b^The model predicting contact with child and adolescent mental health services was adjusted for age, sex, housing tenure, number of stressful life events, impact of mental health difficulties on the child, contact with teacher at follow-up, and contact with primary health care at follow-up

### Secondary analyses

We found no significant interactions between age and gender in predicting access to primary health care (*p* = 0.129) or paediatrics (*p* = 0.250). In contrast, for specialist mental health services, we observed a significant age by gender interaction [*p* = 0.035 (1.13, 27.10)] (see online resource 4). This interaction suggests that, among boys, older participants (≥ 11 years old) have decreased likelihood of reporting access to specialist mental health services as compared to younger participants (aOR = 0.47) while among girls, older participants have increased likelihood of reporting access to specialist services as compared to younger ones (aOR = 2.59). Among younger participants, girls versus boys have decreased likelihood of reporting access to specialist services (aOR = 0.12) and among older participants, girls versus boys also have decreased likelihood of reporting access to specialist services (aOR = 0.66).

## Discussion

We investigated the characteristics of children with pLTCs associated with contact with services in relation to their mental health. Common predictors of contact across the different services were greater impact of difficulties on child’s distress levels and everyday life and contact with the teacher. These findings suggest that services are accessed by children with the greatest mental health needs, in agreement with previous literature showing that the presence or severity of psychopathology is related to contact with different types of services in the UK [10]. In addition, these results highlight the critical role teachers and schools play in the identification of poor mental health and in linking pupils with health care professionals, as demonstrated by a previous UK population study in physically healthier school-aged children [10]. Mental health-related contact with the teacher was consistently the strongest predictor of contact with all three health care services investigated, suggesting that children/families who had been in contact with the teacher were up to nine times more likely to be in contact with a health care professional. This might be an indication of the level of parental or child concern and/or that teachers encourage help-seeking. Interestingly, the majority of participants were rated by teachers to have some or marked educational difficulties in at least one area (reading, spelling, mathematics). This is in line with previous literature showing adolescences who experience worse health to be substantially less likely to complete high school and enter post-secondary education [29]. Conversations with pupils who struggle at school (and/or their parents) may facilitate the identification of mental health symptoms by the teachers who should encourage help-seeking. Educators are, indeed, an easy-accessed, first point of communication for children/families who seek help and, therefore, schools should ensure their staff have access to high quality information so they feel confident to discuss mental health with pupils and their parents.

Predictors specific to primary health care were older age, female gender, larger family size, some or marked academic difficulties, and having parents with educational qualification(s). Adolescent girls are at increased risk of emotional problems compared to younger girls and to boys of all ages [30], while primary health care is traditionally the gate keeper for specialist health care. Ford et al. [10] found a similar interaction between age and gender in relation to mental health-related contact with primary health care professionals among the general population of children. The presence of academic difficulties increased the likelihood of reporting contact with primary health care, which might relate to distress related to struggling at school. Anecdotally, teachers often advise families and young people who are concerned to contact their General Practitioner (Fig. 1), as not all specialist mental health services accept referrals directly from schools. Ford et al. [10] found larger families to be more likely to report contact with paediatrics for mental health advice, rather than primary health care, but larger families may have more frequent contact with General Practitioners, presenting greater familiarity and also opportunities for raising concerns. Finally, parents with educational qualification(s) are likely to have greater health literacy and better access to information, hence greater perception of the need for professional help, and more time and resources to seek it [31].

Predictors specific to contact with child and adolescent mental health services were the experience of more stressful life events over the lifetime and contact with primary health care. Experiencing stressful life events is a known risk factor for poor mental health and has been previously identified as a predictor for contact with child and adolescent mental health services [10]. Contact with primary health care predicted contact with child and adolescent mental health services, replicating the findings of earlier work [10] and in line with the role of frontline services as gate keepers to specialist services. We also observed a significant age by gender interaction on the probability of reporting contact with specialist mental health services, which reflects the age and gender patterns of mental health conditions across childhood and adolescence [32]. More precisely, both younger and older boys were more likely to report contact with mental health services as compared to girls. In addition, younger boys (vs. boys in adolescence) and adolescent girls (vs. younger girls) were more likely to report contact with mental health services. Boys are more likely to develop neurodevelopmental and behaviour problems, which often present during early and mid-childhood, and which may also have a greater impact on themselves and others than emotional problems, which are more frequently seen in adolescent girls. Therefore, mental health disturbances in young boys may be considered as more severe by some parents or teachers who consequently seek specialist help. In line with our findings, a previous UK study has shown that boys in childhood are more likely than girls to be referred to child psychiatrists by general practitioners [33]. Indeed, the way in which psychiatric disorder presents may influence its perceived significance to health professionals and the likelihood of psychiatric referral. Presentations with antisocial behaviours are more likely to precipitate referral than internalising disorders such as depression [34]. Population studies suggest that even when girls meet research diagnostic criteria for neurodevelopmental disorders, they are less likely to be referred and seen by specialist services [35]. Both research, training, and guidelines should be developed that explore how neurodevelopmental conditions present differently in girls to improve their detection and access to treatment.

Surprisingly, paediatric contact did not predict contact with child and adolescent mental health services. Paediatricians may not recognise or underestimate the need for referral to mental health specialists. Previous research suggests that paediatricians only identified a quarter of all cases with possible psychiatric disorder [4], in line with evidence indicating that the mental health problems associated with physical illness in young patients are commonly unrecognised and untreated [18, 36]. Introducing routine mental health screening in paediatric settings could be a beneficial tool for paediatricians to identify children with pLTCs who need specialist interventions [37]. Compared with general paediatricians, paediatric neurologists were more likely to refer children with epilepsy to specialist mental health services and were more cognizant of the benefit from mental health referrals, suggesting that improving paediatricians’ specific knowledge and awareness of the mental health comorbidities of children with pLTCs and the need for referrals is crucial [38]. Alternatively, mental health difficulties may be managed in some cases within paediatric settings because of concerns that referrals may be rejected or pessimism about long waiting lists [39]. Indeed, a recent survey in the UK showed that a sizeable proportion of community paediatricians are involved in the assessment or care of children and young people with mental health difficulties, especially neurodevelopmental conditions, mainly due to difficulty with accessing specialist mental health services [40]. Education to improve the recognition and management of child mental health difficulties should be part of paediatricians training and continued professional development.

Our study highlighted the role of impact as a universal predictor of access to services and suggested that children with mild difficulties are less likely to seek help. Given that child and adolescent specialist mental health services in the UK are under-resourced and over-stretched [41], easy-access, evidence-based interventions should be made available in children with pLTCs who face mental health difficulties. Brief and/or low-intensity (< 6 sessions) psychological interventions based on cognitive behavioural principles [42] have demonstrated the potential to benefit young people with a pLTC [43]. These interventions could be delivered at drop-in centres [44], which could be in primary or secondary health care, or at schools [45]. Our findings also revealed that some groups of children are seen in different health care settings and for different reasons. Each service should be aware of the profile of children it sees to consider which children may currently be under-served and how this situation might be addressed.

Coordination of services across sectors is also crucial. For example, implementing mental health screenings in schools and integrating them with primary health care resources or specialist mental health services could be an efficient and cost-effective way of addressing the mental health needs of CYP. Coordinated home visits by General Practitioners and mental health workers, shift of psychiatric clinics to health care centres, joint consultations and a coordinated management plan including school personnel, primary care clinicians, paediatricians, mental health specialists, and social services in the care of the child, offers the unique opportunity for patient-centred pathways to a holistic care. With the significant shortage of child and adolescent mental health specialists [41], coordinated and shared care with the wider children’s workforce becomes a viable and sustainable way of meeting child mental health needs in the community.

We excluded participants with missing data, but those with missing data were more likely to be more severely affected and to have more risk factors. Multiple imputation was deemed inappropriate in this study since data were not missing at random. Notably, some families declined consent for including teachers in the study which resulted in up to 20% teacher missing data, ultimately reducing the analytic sample size. The exclusion of participants with missing data might have introduced type 2 errors but suggests that the associations we report are likely to be robust. Secondary data analyses are always constrained by the available data. Objective data on severity of symptoms or care need were not available for analysis; future studies should seek to collect these data. We included a relatively small number of participants in the non-white group (5%), which may explain the null associations between ethnicity and service contact. As this was a population-based survey, the assessment of pLTCs was based on parental report which may involve information or recall bias; future research should corroborate these assessments with administrative medical and school data. In addition, we measured the impact of mental health difficulties on child’s distress levels and everyday life using parental report, though difficulties not expressed by the child might go unrecognised by the parent who might consequently not seek help. This suggests that some children with severe symptoms will still be untreated. Future studies need to include children’s report on the impact of mental health difficulties. This study is also limited to examining service contacts for mental health support, but there are no data on the quality of care received by each service, hence contact does not necessarily reflect appropriate problem assessment and management. The provision of care by service are likely to be heterogenous, and therefore different sets of predictors may exist for different types of interventions within services. Future research using administrative data or patient registers should explore this finer level of analysis.

In conclusion, our findings represent a first step towards identifying characteristics that may be barriers or facilitators to accessing mental health care in children with comorbid pLTCs. The role of child-, family- and service-related factors is highlighted, which could inform planning and provision of services to reduce unmet mental health needs in young patients.

### Supplementary Information

Below is the link to the electronic supplementary material.

Supplementary file1 (DOCX 18 KB)

Supplementary file2 (DOCX 22 KB)

Supplementary file3 (DOCX 21 KB)

Supplementary file4 (DOCX 22 KB)

## Data Availability

The data analysed during the current study are available via application to the UK Data Service.
