# Supplementary material for: Predictors of contact with services for mental health problems among children with comorbid long-term physical health conditions: a follow-up study
Source: Eur Child Adolesc Psychiatry. 2022 Nov 10;33(1):21–31. doi: 10.1007/s00787-022-02105-4 (PMC10807016; doi:10.1007/s00787-022-02105-4)
Supplement: Supplementary file 1 — Supplementary file1 (DOCX 18 KB) [file 787_2022_2105_MOESM1_ESM.docx]

Sampling and response

The British Child and Adolescent Mental Health Surveys involved a large, stratified probability sample identified via the child benefit register. The latter was applied to create a sampling frame of postal sectors from Great Britain. At this time, child benefit was a universal benefit and therefore covered all children except those whose residency status was uncertain or temporary, such as children of asylum seekers and members of foreign armed forces. Across the two baseline surveys in 1999 and 2004, approximately 10% of postal sectors were sampled with a probability related to the size of the sector. Postal sectors were stratified by regional health authority and socioeconomic group within it, representing around 90% of all British children once adjusted for inaccurate or ineligible benefit records [1]. Complete mental health assessments were completed with 73% and 65% of the original sample in 1999 and 2004, equating to 83% and 76% of those approached for interview, respectively [2].

All children with a mental health disorder in 1999 (*n* = 911), and a random sample of those without (*n* = 2334), were invited to the follow-up survey three years later. After excluding 9% of participants who were no longer eligible [they had died or had been taken into the care of the local authority], the response rate was 88% (*n* = 2586) of those invited [3]. The 2007 follow-up aimed to include as many of the children who had participated in 2004 as possible, by inviting 90% of them (*n* = 7329) [4]; 8% were lost to follow-up, 12% refused to take part, and 343 children deemed ineligible at follow-up; thus, a final sample of 5326 children retained into the study representing 78% of those approached.

References

1. Stringaris A, Goodman R (2009) Three dimensions of oppositionality in youth. J Child Psychol Psychiatry 50:216-223. https://doi.org/10.1111/j.1469-7610.2008.01989.x
2. Ford T, Vizard T, Sadler K, McManus S, Goodman A, Merad S, et al (2020) Data Resource Profile: Mental Health of Children and Young People (MHCYP) Surveys. Int J Epidemiol 49:363-4g. https://doi.org/10.1093/ije/dyz259
3. Meltzer H, Gatward R, Corbin T, Goodman R, Ford T (2003) Persistence, onset, risk factors and outcomes of childhood mental disorders: TSO, London
4. Parry-Langdon N, Clements A, Fletcher D, Goodman R (2008) Three years on: survey of the development and emotional well-being of children and young people. Office for National Statistics, Newport
