# Supplementary material for: Predictors of contact with services for mental health problems among children with comorbid long-term physical health conditions: a follow-up study
Source: Eur Child Adolesc Psychiatry. 2022 Nov 10;33(1):21–31. doi: 10.1007/s00787-022-02105-4 (PMC10807016; doi:10.1007/s00787-022-02105-4)
Supplement: Supplementary file 2 — Supplementary file2 (DOCX 22 KB) [file 787_2022_2105_MOESM2_ESM.docx]

**Supplementary material**

| Table 1  *Mental health diagnoses at baseline (based on the Development and Wellbeing Assessment) in children and young people (N = 397)* | |
| --- | --- |
| *Separation Anxiety Disorder (n % yes)* | 32 (8.1) |
| *Specific Phobia (n % yes)* | 52 (13.1) |
| *Social Phobia (n % yes)* | 12 (3.0) |
| *Panic Disorder (n % yes)* | 5 (1.3) |
| *Agoraphobia (n % yes)* | 3 (0.8) |
| *Post-traumatic Stress Disorder (n % yes)* | 3 (0.8) |
| *Obsessive-Compulsive Disorder (n % yes)* | 7 (1.8) |
| *Generalised Anxiety Disorder (n % yes)* | 26 (6.5) |
| *Other Anxiety Disorder (n % yes)* | 53 (13.4) |
| *Depressive Episode (n % yes)* | 24 (6.0) |
| *Other Depressive Episode (n % yes)* | 7 (1.8) |
| *Hyperkinesis (n % yes)* | 74 (18.6) |
| *Oppositional Defiant Disorder (n % yes)* | 111 (28.0) |
| *Unsocialised Conduct Disorder (n % yes)* | 23 (5.8) |
| *Socialised Conduct Disorder (n % yes)* | 31 (7.8) |
| *Other Conduct Disorder (n % yes)* | 25 (6.3) |
| *Autistic Spectrum Disorder (n % yes)* | 26 (6.5) |
| *Tic Disorder (n % yes)* | 11 (2.8) |
| *Eating Disorder (n % yes)* | 4 (1.0) |
| ^a^*Selective Mutism (n % yes)* | 1 (0.6) |

*Note*. ^a^ *n* = 156.

N = number; *n* = number.
