# Supplementary material for: Predictors of contact with services for mental health problems among children with comorbid long-term physical health conditions: a follow-up study
Source: Eur Child Adolesc Psychiatry. 2022 Nov 10;33(1):21–31. doi: 10.1007/s00787-022-02105-4 (PMC10807016; doi:10.1007/s00787-022-02105-4)
Supplement: Supplementary file 3 — Supplementary file3 (DOCX 21 KB) [file 787_2022_2105_MOESM3_ESM.docx]

Strengths and Difficulties Questionnaire

| Table 1  *The SDQ questions used in the study* | |
| --- | --- |
| Variables measured | **SDQ question** |
| Parental recognition of mental health difficulties | *Overall, do you think that your child has difficulties in one or more of the following areas: emotions, concentration, behaviour or being able to get on with other people*? |
| Teacher recognition of mental health difficulties | *Overall, do you think that this child has difficulties in one or more of the following areas: emotions, concentration, behaviour or being able to get on with other people*? |
| Impact of mental health difficulties on the child | *Do the difficulties upset or distress your child*  *and*  *Do the difficulties interfere with your child's everyday life in the following areas: home life, friendships, classroom learning, leisure activities*? |
| Burden on the family | *Do the difficulties put a burden on you or the family as a whole*? |
| *Note*. SDQ = Strengths and Difficulties Questionnaire. | |
