# Supplementary material for: Predictors of contact with services for mental health problems among children with comorbid long-term physical health conditions: a follow-up study
Source: Eur Child Adolesc Psychiatry. 2022 Nov 10;33(1):21–31. doi: 10.1007/s00787-022-02105-4 (PMC10807016; doi:10.1007/s00787-022-02105-4)
Supplement: Supplementary file 4 — Supplementary file4 (DOCX 22 KB) [file 787_2022_2105_MOESM4_ESM.docx]

**Fig 1**. Age by sex interaction on the probability of contact with child and adolescent mental health services, adjusting for covariates. Error bars indicate 95% confidence intervals
